# Histone lactylation-induced GLI3 activation drives macrophage M1 polarization and exosomal SERPINE1 release in abdominal aortic aneurysm progression

**DOI:** 10.1038/s41420-025-02748-7

**Published:** 2025-11-10

**Authors:** Kai Yuan, Junjian Liu, Guanghui Chen, Peng Wang, Liang Wei

**Affiliations:** 1 Department of Vascular Surgery, Renji Hospital, School of Medicine, Shanghai Jiao Tong University, Shanghai, China; 2 The third department of surgery, Dayao People’s Hospital, Yunnan, China

**Keywords:** Aneurysm, Mechanisms of disease

## Abstract

M1 macrophages promote the progression of abdominal aortic aneurysm (AAA). Lactate-mediated histone lactylation modification regulates macrophage M1 polarization. However, the role and molecular mechanisms of lactylation-mediated macrophage M1 polarization in AAA remain unclear. Histone lactylation in macrophages was investigated using clinical specimens and AAA animal models, with subsequent validation at the cellular level. Key genes undergoing lactylation modification were screened through RNA-seq and CUT&Tag joint analysis. Functional validation was conducted to demonstrate that the target genes mediate AAA progression by regulating macrophage M1 polarization and subsequent exosome-mediated endothelial dysfunction. We observed elevated serum lactate levels in AAA patients. H3K18 lactylation (H3K18la) and M1 macrophage marker CD86 were upregulated and co-localized in the aortic aneurysm of AAA mice. Lactate upregulated H3K18la levels and M1 polarization markers CD80, MCP-1, and iNOS, while downregulating M2 polarization marker CD206. Transcriptome sequencing identified 305 genes that were abnormally expressed in lactate-treated macrophages. A total of 839 differentially modified lactylation peaks were identified in lactate-treated macrophages, with 195 peaks enhanced and 644 peaks weakened. Notably, GLI3 was identified as a highly lactylated gene, and its knockdown significantly suppressed macrophage M1 polarization. Lactate induced macrophage M1 polarization by promoting GLI3 expression. Furthermore, lactate-polarized M1 macrophages induced endothelial dysfunction by secreting the exosomal protein SERPINE1. This study demonstrates that histone lactylation-mediated GLI3 activation drives macrophage M1 polarization and subsequent secretion of exosomal SERPINE1, inducing endothelial dysfunction in AAA progression. This study provides significant advances in our comprehensive understanding of AAA pathogenesis mediated by endothelial dysfunction.

## Introduction

Abdominal aortic aneurysm (AAA) is characterized by a persistent and localized enlargement of the abdominal aorta, surpassing 50% of the normal vessel diameter or measuring more than 3 cm in diameter [1]. The main pathological features of AAA include weakened and dilated abdominal aorta and chronic inflammation [2]. AAA-related aortic rupture leads to ~200,000 deaths worldwide each year [3]. Presently, apart from surgical treatment, there are no specific drugs available to effectively prevent abdominal aortic dilation or rupture. However, the cellular and molecular mechanisms underlying the etiology and progression of AAA are still not fully understood, impeding advancements in drug development and clinical treatment in this field.

Macrophages have a pivotal role in the pathogenesis of AAA [4–6]. Macrophages can be polarized into M1 macrophages and M2 macrophages [7]. M1 macrophages exacerbate local inflammation and promote aortic dilation and vascular remodeling by upregulating a plethora of inflammatory cytokines [5]. A study has shown that circRNA Cdyl facilitates the development of AAA by inducing macrophage M1 polarization and M1-type inflammation [5]. Elastin-derived peptide promotes AAA formation by regulating the balance of M1/M2 macrophages [4]. Conventional T cells enhance the polarization of M1-like macrophages by secreting granulocyte-macrophage colony-stimulating factor, thereby exacerbating the progression of AAA [6]. However, the precise mechanisms that govern macrophage M1 polarization in AAA remain incompletely understood.

Histone lactylation is a post-translational modification of histones that participates in important biological activities such as macrophage polarization, glycolysis-related cellular functions, and neural system regulation [8]. Lactylation of histone lysine residues mediated by lactate promotes macrophage M1 polarization and regulates gene expression and cellular metabolism [9]. In the later stages of macrophage M1 polarization, the increased production of lactate leads to histone lactylation, which in turn regulates the induction of an M2-like phenotype, promoting wound healing [10]. These studies demonstrate that lactylation modification regulates M1 macrophage polarization. However, there have been no reports on the role of lactylation modifications in AAA. Whether lactylation modifications are involved in AAA progression through the regulation of M1 polarization is also still unclear.

Exosomes serve as critical mediators of intercellular communication [11]. While exosomes derived from M2 macrophages promote angiogenesis and improve cardiac function post-myocardial infarction [12], those from M1-polarized macrophages suppress vascular growth and exacerbate cardiac dysfunction [13]. Notably, lactate promotes macrophage HMGB1 lactylation in polymicrobial sepsis, inducing endothelial dysfunction (an important indicator in AAA) through the secretion of HMGB1 via exosomes [14]. However, the role and mechanism of lactate-induced histone lactation of macrophages in endothelial dysfunction through exosomes are not fully understood.

The objective of this study was to elucidate the role and molecular mechanisms of lactate-mediated histone lactylation in regulating macrophage M1 polarization in AAA. We validated the regulation of histone lactylation and macrophage M1 polarization by lactate through cellular and animal experiments. By integrating transcriptomic sequencing and H3K18la cleavage under target & tagmentation (CUT&Tag), we identified key genes involved in regulating AAA and exhibited high histone lactylation modification. This study establishes a theoretical foundation for the development of drugs and clinical interventions targeting AAA.

## Results

### Histone lactylation was elevated in M1 macrophages of AAA

**Fig. 1:**
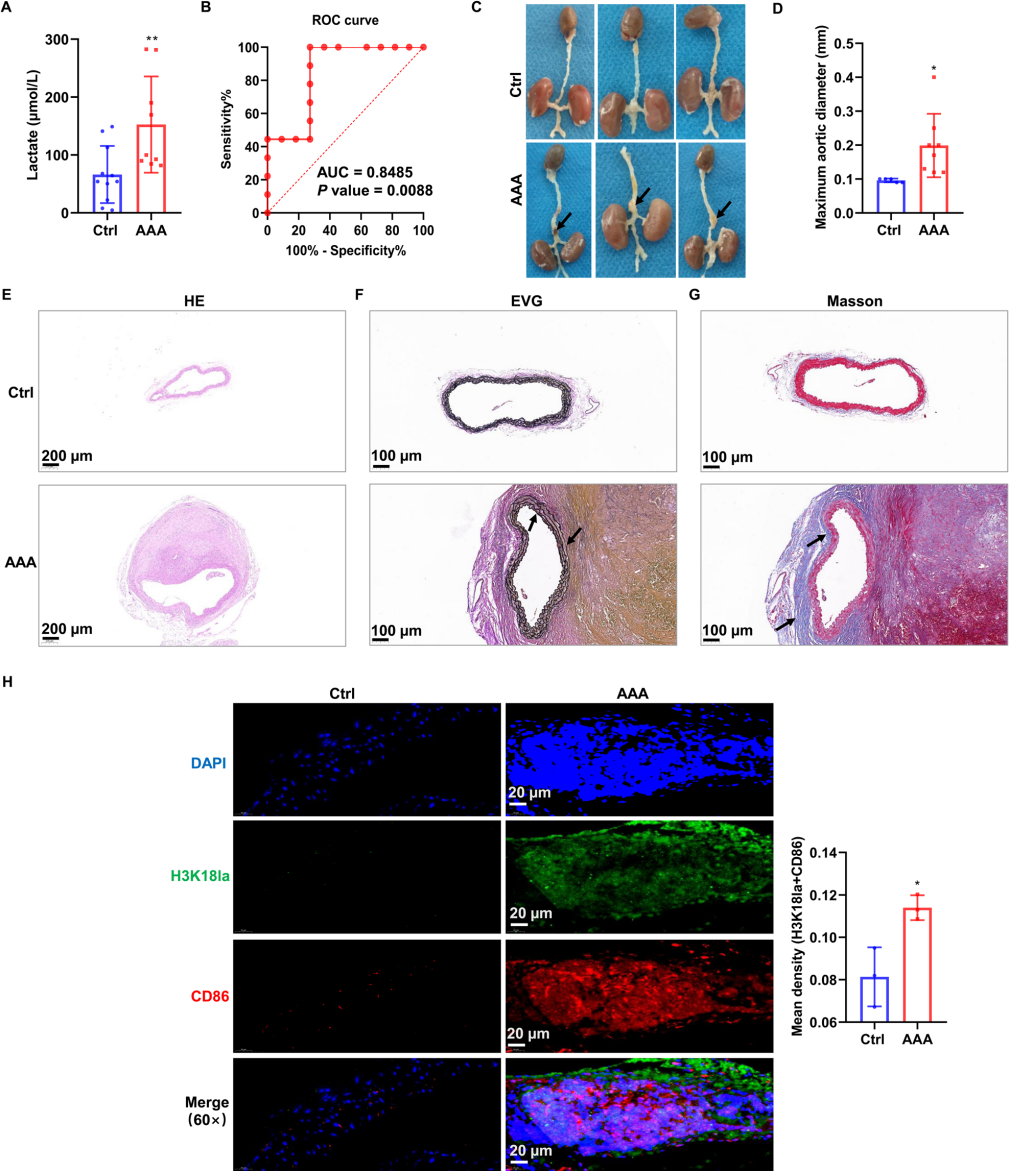
Elevated levels of histone lactylation in macrophages were observed in AAA. **A** The content of serum lactate in AAA patients was detected (control, *N* = 11; AAA, *N* = 9). **B** The ROC curve showed the sensitivity and specificity of lactate in AAA patients. **C** The aorta of mice was shown in the photographs. The black arrow indicates an AAA. **D** Statistical analysis was performed on the maximum arterial diameter in mice (control, *N* = 5; AAA, *N* = 10. Two mice in the AAA group died during the experiment. **E**–**G** Representative images of H&E, EVG, and Masson’s trichrome staining were presented to evaluate vascular structural alterations (*N* = 3). Black arrows in (**F**) indicate disrupted elastic fibers, and black arrows in (**G**) denote fibrotic areas. **H** The IF was used to examine the levels of H3K18la and CD86 in aortic tissue (*N* = 3). Data are represented as mean ± SEM. Two-tailed Student’s *t* test (A and D). *P < 0.05, **P < 0.01.

To explore the impact of lactylation modification on the regulation of AAA, we collected serum samples from AAA patients and healthy volunteers and measured lactate levels. The results showed a notable elevation in serum lactate levels among AAA patients in comparison to the control group (Fig. 1A). The receiver operating characteristic (ROC) curve was constructed for lactate, which revealed an area under the curve (AUC) of 0.8485 (*P* = 0.0088). The diagnostic performance of lactate for AAA was characterized by a sensitivity of 88.89% and a specificity of 72.73% (Fig. 1B). Based on these results, it can be inferred that lactate has the potential to function as a diagnostic biomarker for AAA. To further investigate whether histone lactylation is increased in AAA, we established an AAA mouse model by injection of Ang II. The AAA group exhibited a significant elevation in the maximum arterial diameter compared to the control group (Fig. 1C, D). Histopathological changes were examined using H&E, EVG, and Masson’s trichrome staining. Compared with the NC group, mice in the AAA group exhibited marked abdominal aortic dilation, reduced collagen content, and increased fragmentation of elastic fibers (Fig. 1E–G). Furthermore, we assessed the histone lactylation level and the expression of the M1 macrophage marker CD86 in abdominal aortic tissue. The IF analysis revealed upregulated levels of H3K18la and increased CD86 expression in the AAA group, and H3K18la co-localized with CD86 in the aortic aneurysm, suggesting both an expansion of M1 macrophages and enhanced H3K18la levels within these M1 macrophages during AAA pathogenesis (Fig. 1H). These results suggeste that lactate levels are elevated in AAA and that there is an upregulation of histone lactylation levels in M1 macrophages.

### Lactate induces histone lactylation modification and M1 polarization in macrophages

**Fig. 2:**
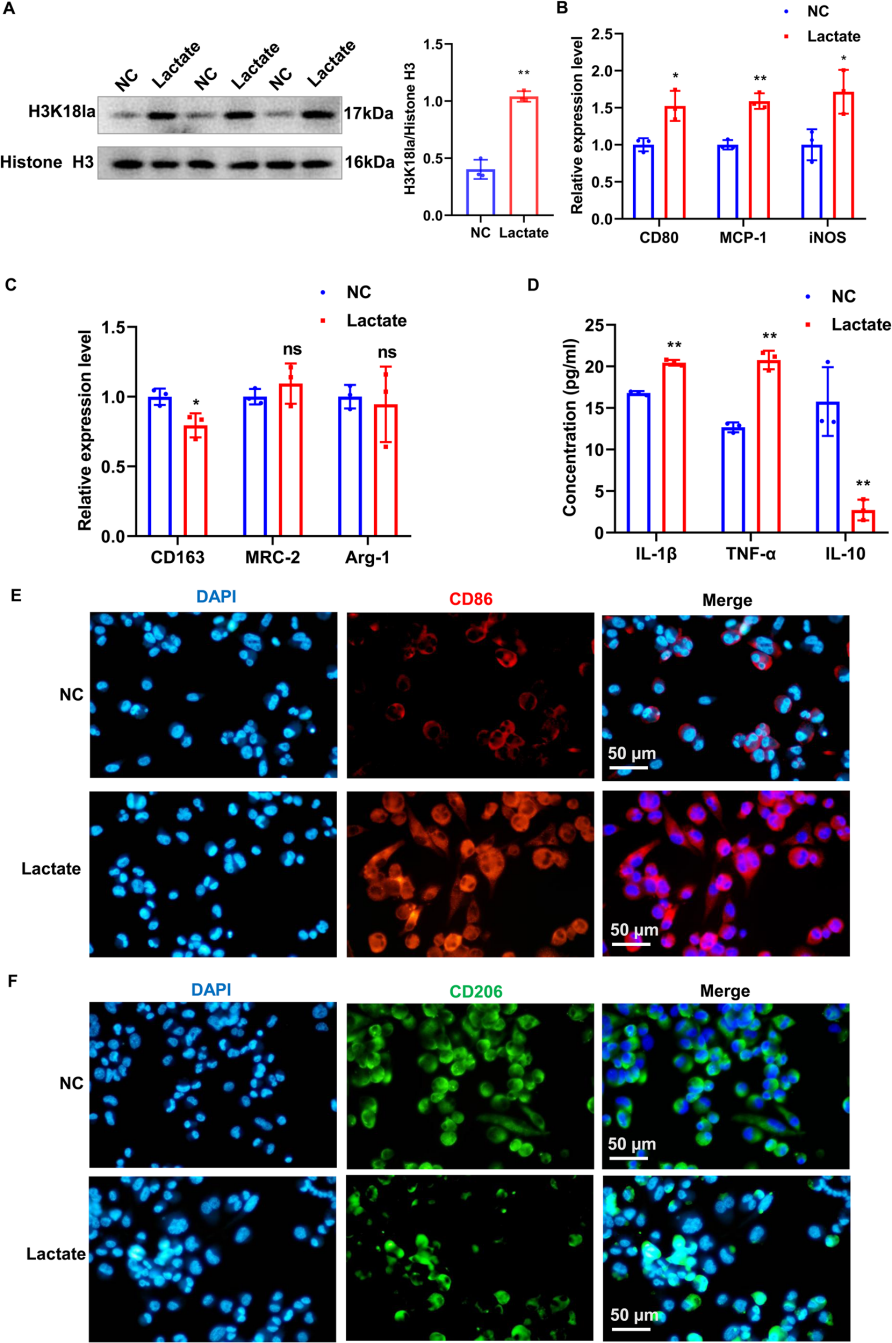
The macrophages’ M1 polarization and histone lactylation were induced by lactate. **A** The levels of H3K18la in macrophages from the control group and lactate-treated group were detected by WB. **B**, **C** The expression of M1 polarization marker genes CD80, MCP-1, and iNOS and M2 polarization marker genes CD163, MRC-2, and Arg-1 was measured by RT-qPCR. **D** The inflammatory cytokines IL-10, TNF-α, and IL-1β were assessed using ELISA. **E**, **F** IF was employed to examine the expression of CD86 and CD206. ns: no significant difference. Data are represented as mean ± SEM. Two-tailed Student’s *t* test (**A**–**D**). *P < 0.05, ** *P* < 0.01.

We conducted cellular experiments to validate the induction of macrophage M1 polarization and histone lactylation by lactate in AAA. THP-1 cells were stimulated with PMA in vitro to induce their differentiation into macrophages, followed by lactate treatment. We assessed the level of H3K18la in the cells through WB, and the results revealed a significant increase in H3K18la modification after lactate treatment (Fig. 2A). The expression levels of polarization markers were examined, and the RT-qPCR results demonstrated a significant upregulation of M1 markers CD80, MCP-1, and iNOS in the lactate-treated group (Fig. 2B), while the M2 marker CD163 was significantly downregulated, and there were no significant changes observed in MRC-2 and Arg-1 (Fig. 2C). The levels of inflammatory cytokines were measured using ELISA, and we found that lactate treatment significantly upregulated TNF-α and IL-1β levels, while IL-10 was significantly downregulated (Fig. 2D). IF of macrophage polarization markers revealed an upregulation of the M1 marker CD86 after lactate treatment (Fig. 2E), while the M2 polarization marker CD206 was downregulated (Fig. 2F). These findings suggeste that lactate induces histone lactylation and macrophage M1 polarization.

### Transcriptome sequencing reveals dysregulated gene expression in lactate-treated macrophages

**Fig. 3:**
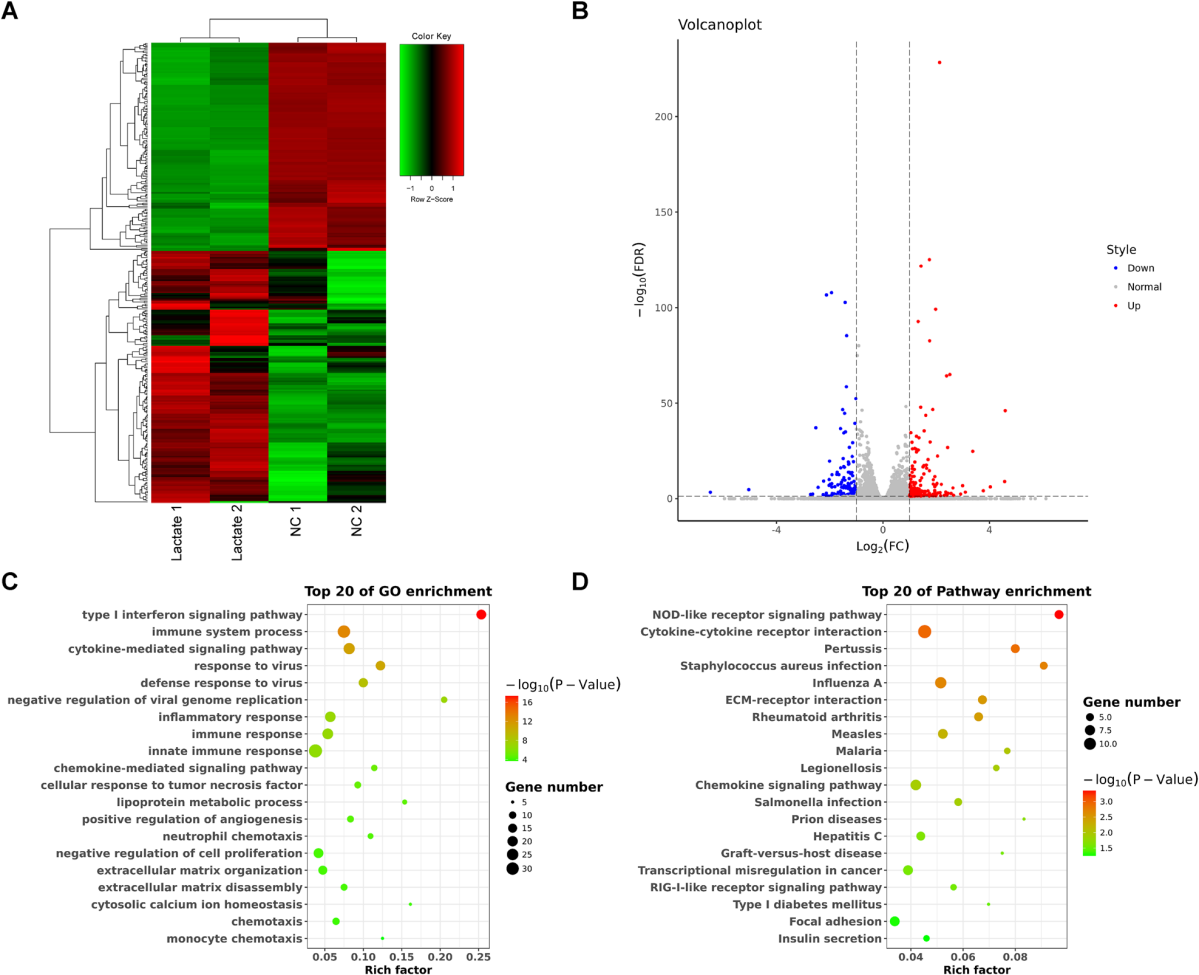
The gene expression profiling analysis was performed in lactate-treated macrophages. **A** The heatmap displayed the changes in gene expression in macrophages following lactate treatment. **B** The DEGs in macrophages were exhibited in the volcano plot (|log2FC | > 1, FDR < 0.05). **C** The enriched terms in the GO analysis of the DEGs in macrophages are visualized in the bubble plot. **D** KEGG analysis showed pathways associated with the DEGs in macrophages.

To elucidate the underlying molecular mechanisms of lactate regulation of M1 polarization in macrophages, we collected PBMCs from healthy human blood. PBMCs were induced into macrophages using M-CSF and incubated with lactate (10 mM). Subsequently, transcriptome sequencing was performed on lactate-treated and untreated macrophages. The results revealed a total of 18,725 genes that were successfully mapped, among which 305 genes exhibited differential expression. In comparison to the control group, the lactate-treated group exhibited an increase in the expression of 167 genes and a decrease in the expression of 138 genes (|log2FC | > 1, FDR < 0.05) (Fig. 3A, B). GO analysis of the DEGs indicated their enrichment in GO terms such as type I interferon signaling pathway, immune system processes, and cytokine-mediated signaling pathway (Fig. 3C). Pathway analysis further revealed that the DEGs were enriched in pathways including the NOD-like receptor signaling pathway and Chemokine signaling pathway (Fig. 3D). These results indicate that there is dysregulation of transcriptional expression in lactate-treated macrophages.

### CUT&Tag reveals abnormal histone lactylation modification in lactate-treated macrophages

**Fig. 4:**
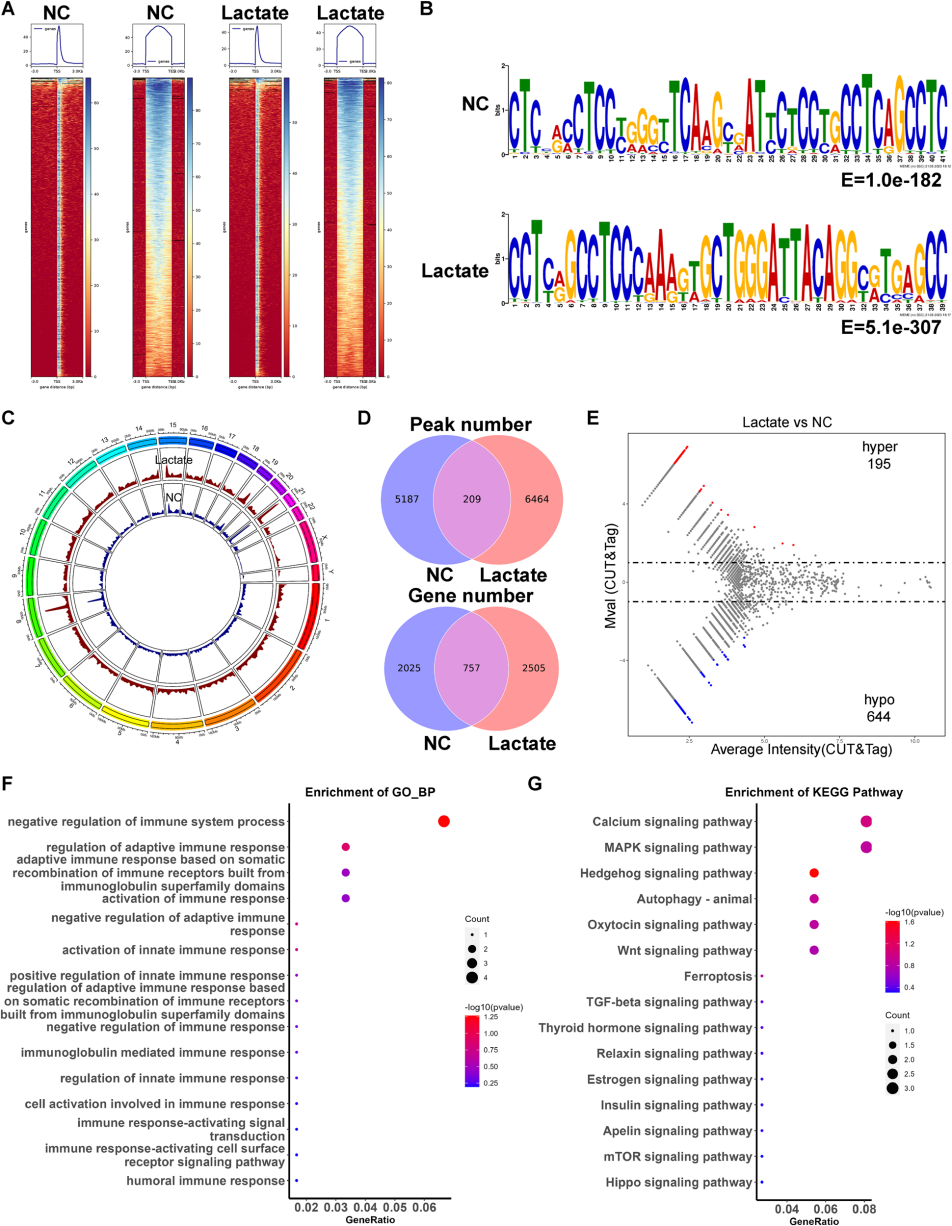
CUT&Tag reveals abnormal histone lactylation modification in lactate-treated macrophages. **A** The heatmap showed the H3K18la signals in macrophages. **B** Motif analysis was conducted on the sites of histone lactylation modification. **C** The circos plot displayed the genomic location information of differentially lactylated genes identified by CUT&Tag. **D** The number of peaks (left) and genes (right) with histone lactylation modification in macrophages. **E** The MA plot illustrated the number of differential peaks of lactylation modification between the NC group and the lactate-treated group (|*M* value | > 1, *P* < 0.03). **F** GO analysis was performed on the genes with upregulated lactylation modification levels. **G** The enrichment pathways for the genes with upregulated lactylation modification levels were identified by KEGG.

To determine the changes in histone lactylation in AAA, we collected PBMCs from the blood of healthy individuals and induced PBMCs into macrophages using M-CSF, followed by treatment with lactate (10 mM). The macrophages were subjected to CUT&Tag using H3K18la antibody. We found that the enrichment of the H3K18la peak was increased in lactate-treated macrophages compared to controls (Fig. 4A). Motif analysis of all histone lactylation modification peaks showed that the predominant motif of the control group was CTCCxCCTCCxGGGTTCAAGCGATTCTCCTGCCTCAGCCTC, while the predominant motif in the lactate-treated group was CCTCxGCCTCCCAAAGTGCTGGGATTACAGGCGTGAGCC (Fig. 4B). Furthermore, we observed differences in histone lactylation modification peaks between the lactate-treated group and the control group (Fig. 4C). A total of 209 peaks were identified as common peaks. The control group had 5187 unique peaks, and the lactate group had 6464 unique peaks (Fig. 4D, up). Among the genes associated with the modified peaks, 757 genes were identified as common genes. The control group had 2025 unique genes, and the lactate group had 2505 unique genes (Fig. 4D, down). Next, we identified differential peaks of lactylation modification following lactate treatment. The results revealed 839 differential peaks, with 195 peaks enhanced and 644 peaks weakened (|M.value | > 1, *P* < 0.03) (Fig. 4E). GO analysis of the genes with upregulated histone lactylation levels revealed their involvement in the regulation of adaptive immune response and activation of immune response (Fig. 4F). KEGG revealed an enrichment of genes with increased histone lactylation in pathways such as Hedgehog signaling pathway, Autophagy-animal, and Wnt signaling pathway (Fig. 4G). These data indicate that there is an abnormal landscape of histone lactylation modification in macrophages after lactate treatment.

### CUT&Tag and transcriptome sequencing identify COL27A1, SLFNL1, GLI3, SEMA5A, and RPS3AP5 as key genes regulated by histone lactylation modification in macrophages

**Fig. 5:**
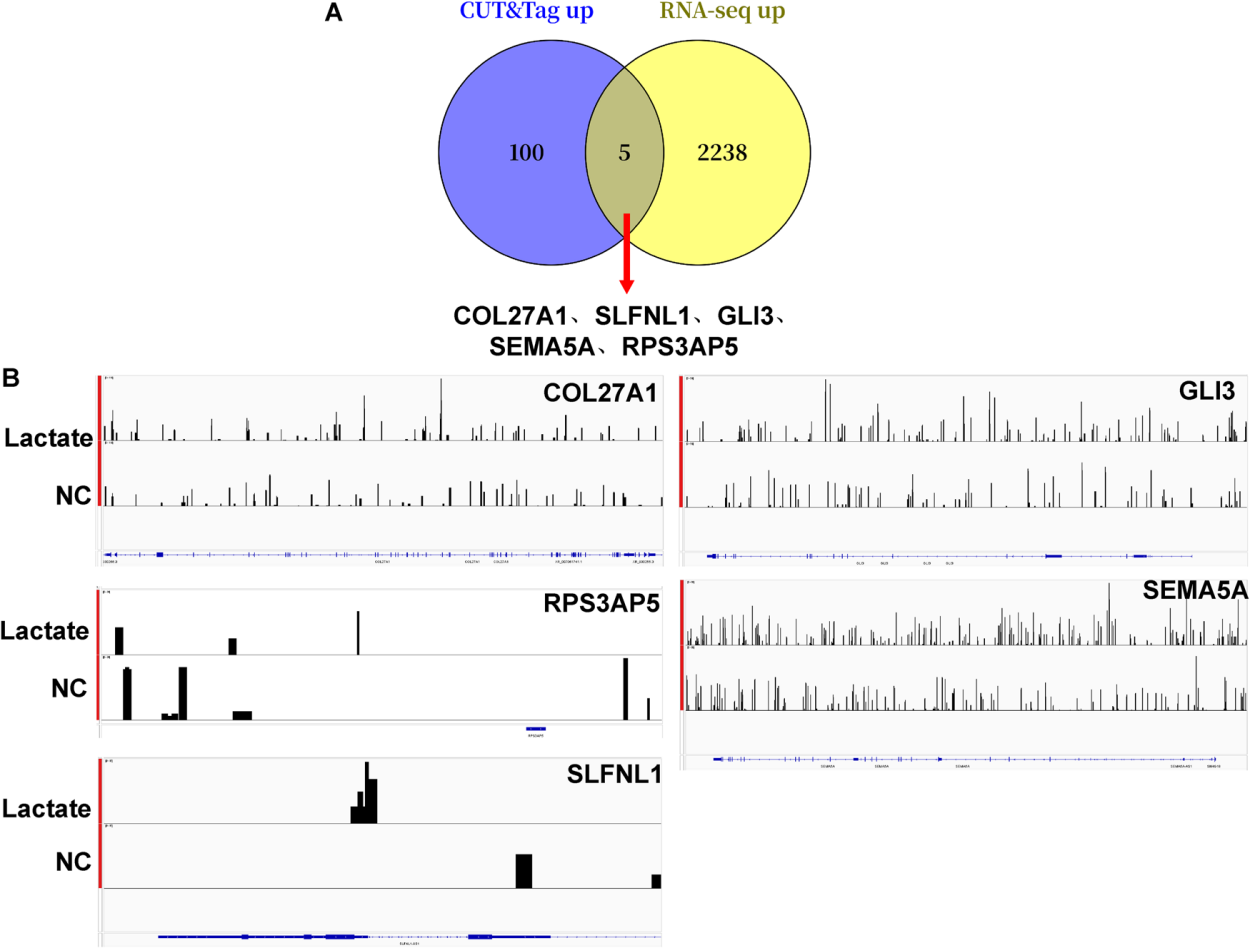
Transcriptome sequencing and CUT&Tag were combined to screen for key genes undergoing differential lactylation modification in lactate-treated macrophages. **A** A Venn diagram illustrates the intersection of genes identified by transcriptome sequencing and CUT&Tag. **B** The lactylation modification peak graphs of the five selected genes were obtained.

Next, we further identify the key target genes mediated by lactylation modification in lactate-treated macrophages. Given that histone lactylation is upregulated in lactate-treated macrophages, and it has been shown that lactylation promotes transcriptional activation of genes [15], we intersected the upregulated DEGs from transcriptome sequencing and the genes exhibiting high lactylation modification in CUT&Tag. A total of five intersected genes were obtained, including COL27A1, SLFNL1, GLI3, SEMA5A, and RPS3AP5 (Fig. 5A). Visual analysis of the lactylation modification peaks of the five genes revealed that the lactylation peak signal intensity was stronger after lactate treatment (Fig. 5B). These findings suggeste that COL27A1, SLFNL1, GLI3, SEMA5A, and RPS3AP5 are key genes that exhibit high histone lactylation modification in macrophages.

### Histone lactylation-mediated GLI3 induces macrophage M1 polarization

**Fig. 6:**
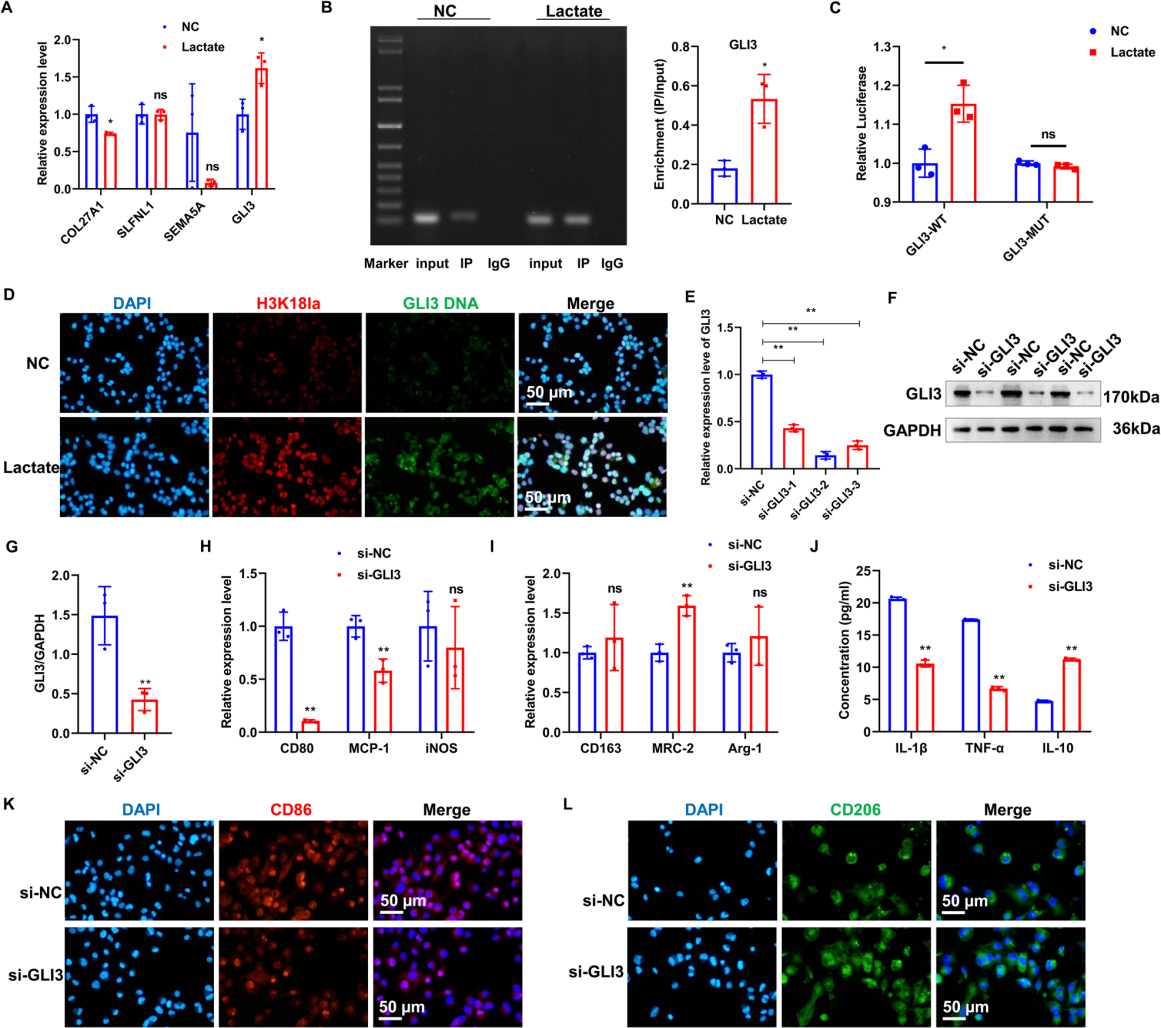
The key gene GLI3, regulated with histone lactylation modification, promotes macrophage M1 polarization. **A** The expression of COL27A1, SLFNL1, SEMA5A, and GLI3 in macrophages was assessed by RT-qPCR. **B** ChIP-qPCR with anti-H3K18la antibody was performed to assess changes in histone lactylation levels at the GLI3 locus in macrophages following lactate treatment. **C** A dual-luciferase reporter assay was conducted to verify the enrichment sites of histone lactylation. **D** IF using an anti-H3K18la antibody combined with FISH targeting GLI3 DNA was carried out to demonstrate the colocalization of histone lactylation and GLI3 DNA in macrophages. **E**–**G** The interference efficiency of GLI3 was detected using RT-qPCR (**E**) and WB (**F**–**G**). **H** The level of CD80, MCP-1, and iNOS after GLI3 knockdown was evaluated using RT-qPCR. **I** The effect of GLI3 on the expression of M2 polarization markers CD163, MRC-2, and Arg-1 in macrophages was investigated by RT-qPCR. **J** The levels of IL-10, TNF-α, and IL-1β after GLI3 knockdown were detected using ELISA. **K**, **L** IF was employed to examine the expression of CD86 and CD206 after GLI3 knockdown. Data are represented as mean ± SEM. Two-tailed Student’s *t* test (**A**–**C** and **G**–**J**) or one-way ANOVA (**E**). *P < 0.05, ** *P* < 0.01.

To further identify macrophage polarization-related genes regulated by histone lactylation, we validated the expression of the candidate genes. Notably, RPS3AP5 was excluded from analysis as its transcript could not be identified in the NCBI database. RT-qPCR analysis revealed that COL27A1 expression was significantly downregulated following lactate treatment, while SLFNL1 and SEMA5A showed no significant changes (Fig. 6A). Importantly, GLI3 expression was markedly upregulated, which is consistent with the transcriptome sequencing results (Fig. 6A). Additionally, CUT&Tag profiling identified lactylation-modified loci in the intronic sequences of the GLI3 gene. GLI3 is a crucial transcription factor in the Hedgehog pathway, which has been implicated in the regulation of macrophage polarization [16–18]. Therefore, we selected GLI3 for further investigation. Macrophages were treated with lactate, and ChIP-qPCR was performed using an H3K18la-specific antibody to assess the histone lactylation status associated with GLI3. Compared with the NC group, lactate treatment significantly enhanced histone lactylation at the GLI3 region (Fig. 6B). To further confirm the regulatory effect of lactate on GLI3 lactylation, a luciferase reporter assay was conducted using constructs containing either the wild-type GLI3 (GLI3-WT) or a mutant lacking the H3K18la modification site (GLI3-MUT). Lactate treatment significantly increased luciferase activity in the GLI3-WT group, while no such effect was observed in the GLI3-MUT group (Fig. 6C). Moreover, IF (H3K18la antibody) combined with FISH (GLI3 DNA probe) demonstrated colocalization of H3K18la and GLI3 DNA in macrophages (Fig. 6D). These findings indicate that lactate upregulates GLI3 expression in macrophages through H3K18la-mediated transcriptional activation.

To clarify the impact of GLI3 on macrophage M1 polarization, we knocked down GLI3 in macrophages and incubated them with lactate. Compared to the control group, all three siRNA knockdown groups showed a significant decrease in GLI3 expression (Fig. 6E). WB results also demonstrated a significant knockdown efficiency of GLI3 (Fig. 6F, G). Then, we examined the levels of macrophage polarization markers and found that knockdown of GLI3 significantly downregulated the M1 markers iNOS, MCP-1, and CD80 (Fig. 6H). The M2 marker MRC-2 was significantly downregulated, while CD163 and Arg-1 showed no significant changes (Fig. 6I). ELISA results indicated that GLI3 knockdown resulted in a decrease of TNF-α and IL-1β, and a significant increase of IL-10 (Fig. 6J). The IF results indicated that knockdown of GLI3 reduced CD86 expression (Fig. 6K) and increased CD206 expression (Fig. 6L). These findings suggeste that knockdown of GLI3 hinders the macrophage polarization towards the M1 phenotype.

### Lactate induces macrophage M1 polarization via GLI3

**Fig. 7:**
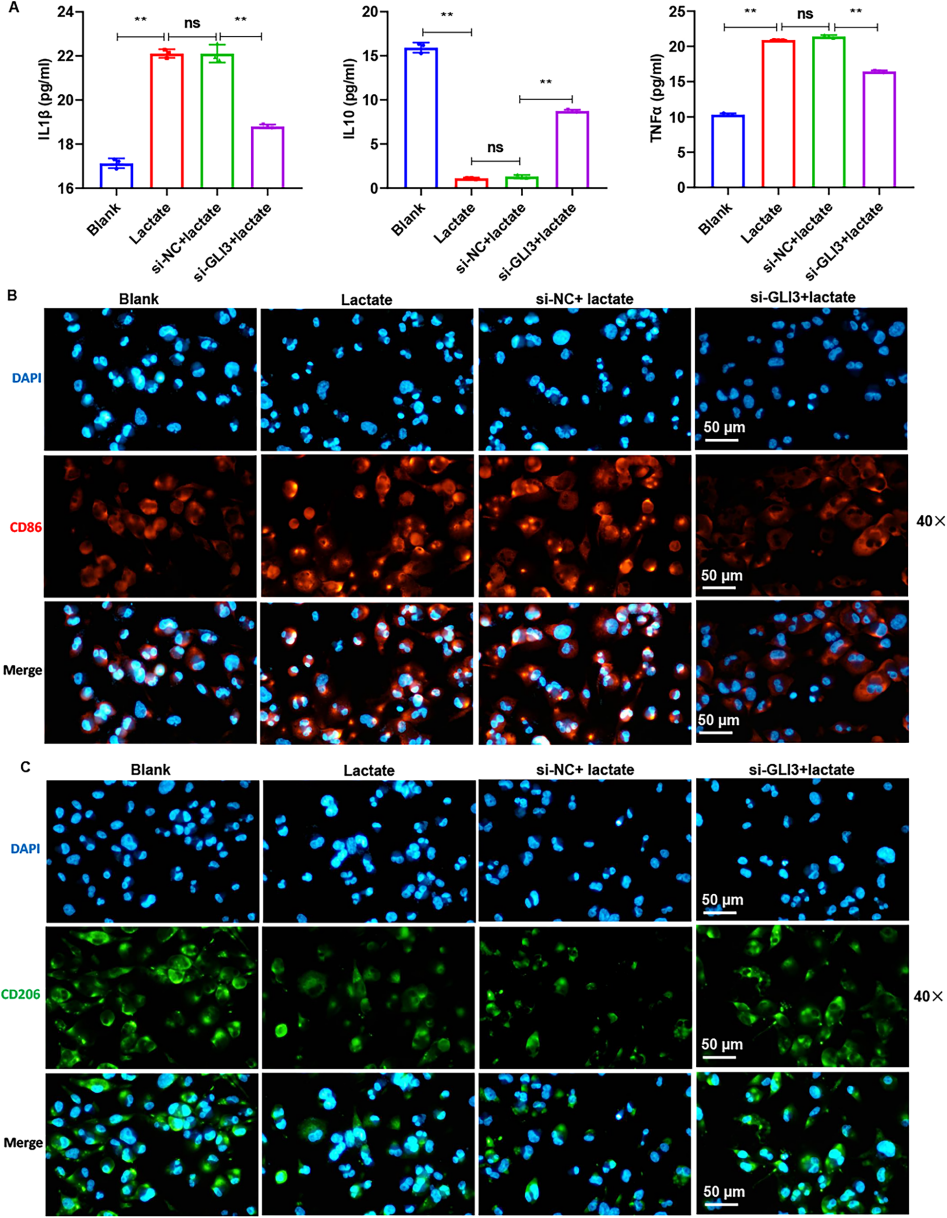
The regulatory effect of lactate on macrophage M1 polarization through GLI3 was confirmed through rescue experiments. **A** ELISA was used to assess the regulation of GLI3 knockdown and lactate treatment on IL-1β, IL-10, and TNF-α in macrophages. **B**, **C** The impact of GLI3 knockdown and lactate treatment on CD206 and CD86 was assessed by IF. Data are represented as mean ± SEM. One-way ANOVA (**A**). ** *P* < 0.01.

To validate the impact of lactate on promoting M1 polarization of macrophages through inducing GLI3 expression, we conducted rescue experiments. ELISA analysis of inflammatory cytokine levels showed that GLI3 knockdown significantly reversed the lactate-mediated upregulation of TNF-α and IL-1β, as well as the downregulation of IL-10 (Fig. 7A). IF results revealed that knockdown of GLI3 partially counteracted the lactate-induced upregulation of CD86 and downregulation of CD206 (Fig. 7B, C). These findings indicate that lactate promotes macrophage M1 polarization through a GLI3-dependent mechanism.

### Exosomes derived from lactate-treated M1 macrophages induce functional impairment of vascular endothelial cells

**Fig. 8:**
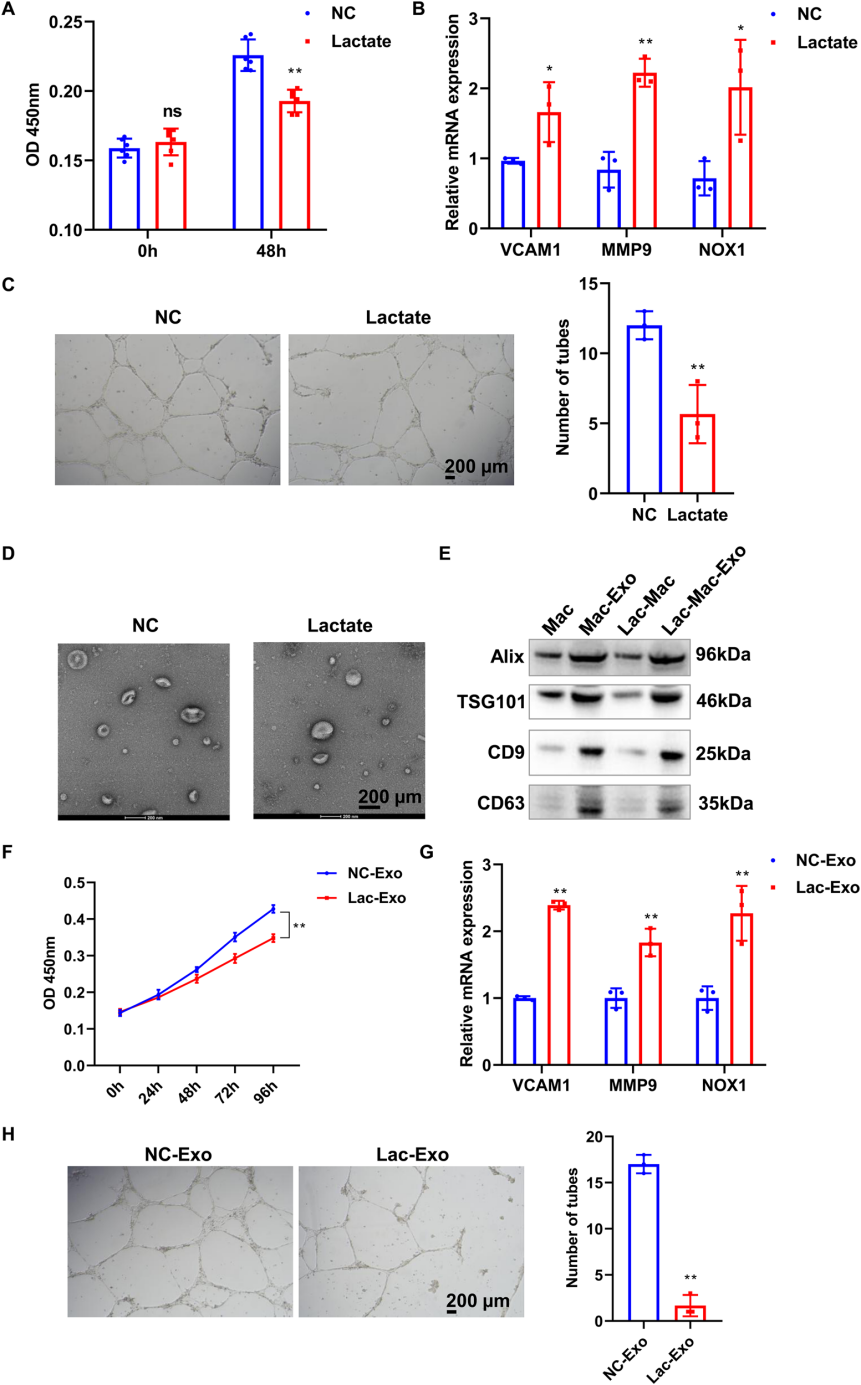
Exosomes derived from lactate-treated macrophages induce endothelial cell dysfunction. **A** Viability of HUVEC was assessed using the CCK-8 assay after co-culture with lactate-treated macrophages. **B** RT-qPCR was employed to detect the endothelial cell dysfunction markers VCAM1, MMP9, and NOX1. **C** The tube formation assay was employed to assess the influence of macrophages on the tube formation of HUVEC. **D** Representative electron micrographs of exosomes isolated from macrophages. **E** Exosomal markers Alix, TSG101, CD9, and CD63 were analyzed in isolated exosomes and whole cell lysates from macrophages by WB. **F** HUVECs were incubated with exosomes isolated from control and lactate-treated macrophages, and cell viability was assessed by CCK-8 assay. **G** mRNA expression levels of VCAM1, MMP9, and NOX1 in exosome-treated HUVECs were determined by RT-qPCR. **H** The impact of macrophage-derived exosomes on HUVEC tube formation capacity was evaluated using an in vitro angiogenesis assay. Data are represented as mean ± SEM. Two-tailed Student’s *t* test (**A**–**C** and **F**–**H**). *P < 0.05, ** *P* < 0.01.

M1 macrophage-mediated inflammatory response is a key factor leading to endothelial cell dysfunction, which is an early pathological event in the formation of AAA [19, 20]. However, the effects of lactate-induced M1 macrophages on endothelial cell dysfunction remain unclear. To investigate whether lactate-induced M1 macrophages regulate endothelial cell function, we co-cultured macrophages with HUVEC after lactate treatment. The CCK-8 revealed that the co-culture of lactate-treated macrophages with HUVEC inhibited the viability of HUVEC compared to the control group (Fig. 8A). The expression of endothelial dysfunction markers VCAM1, MMP9, and NOX1 in the lactate-treated group was significantly elevated, as indicated by the RT-qPCR (Fig. 8B). Furthermore, we assessed the impact of lactate-treated macrophages on the tube formation capacity of HUVEC. Angiogenesis is critical for vascular repair, and its impairment exacerbates endothelial dysfunction in AAA pathogenesis [21]. We found that lactate-induced M1 macrophages significantly suppressed the tube formation ability of HUVEC (Fig. 8C). These results suggest that lactate-induced M1 macrophages induce functional impairment of vascular endothelial cells.

Next, we investigated the molecular mechanisms underlying the crosstalk between M1 macrophages and endothelial cells. Based on established evidence that macrophage-derived exosomes regulate endothelial function [22], we hypothesized whether exosomal communication mediates endothelial dysfunction induced by lactate-polarized M1 macrophages. Therefore, exosomes derived from lactate-treated macrophages were extracted and used to incubate HUVECs. TEM confirmed the characteristic cup-shaped morphology of the isolated exosomes (Fig. 8D). WB analysis demonstrated significant enrichment of exosomal markers, including Alix, TSG101, CD9, and CD63, in the exosome fraction compared to whole macrophage lysates (Fig. 8E). CCK-8 results demonstrated that exosomes isolated from lactate-treated macrophages significantly suppressed HUVEC proliferation compared with exosomes in the control group (Fig. 8F) and upregulated key pathological markers VCAM1, MMP9, and NOX1 (Fig. 8G). Functional assessment revealed that HUVECs incubated with exosomes derived from lactate-treated macrophages exhibited substantial impairment of tube formation capacity (Fig. 8H). These results suggest that exosomes secreted by M1 macrophages induce endothelial dysfunction.

### Exosomal SERPINE1 from M1 macrophages mediates endothelial dysfunction

**Fig. 9:**
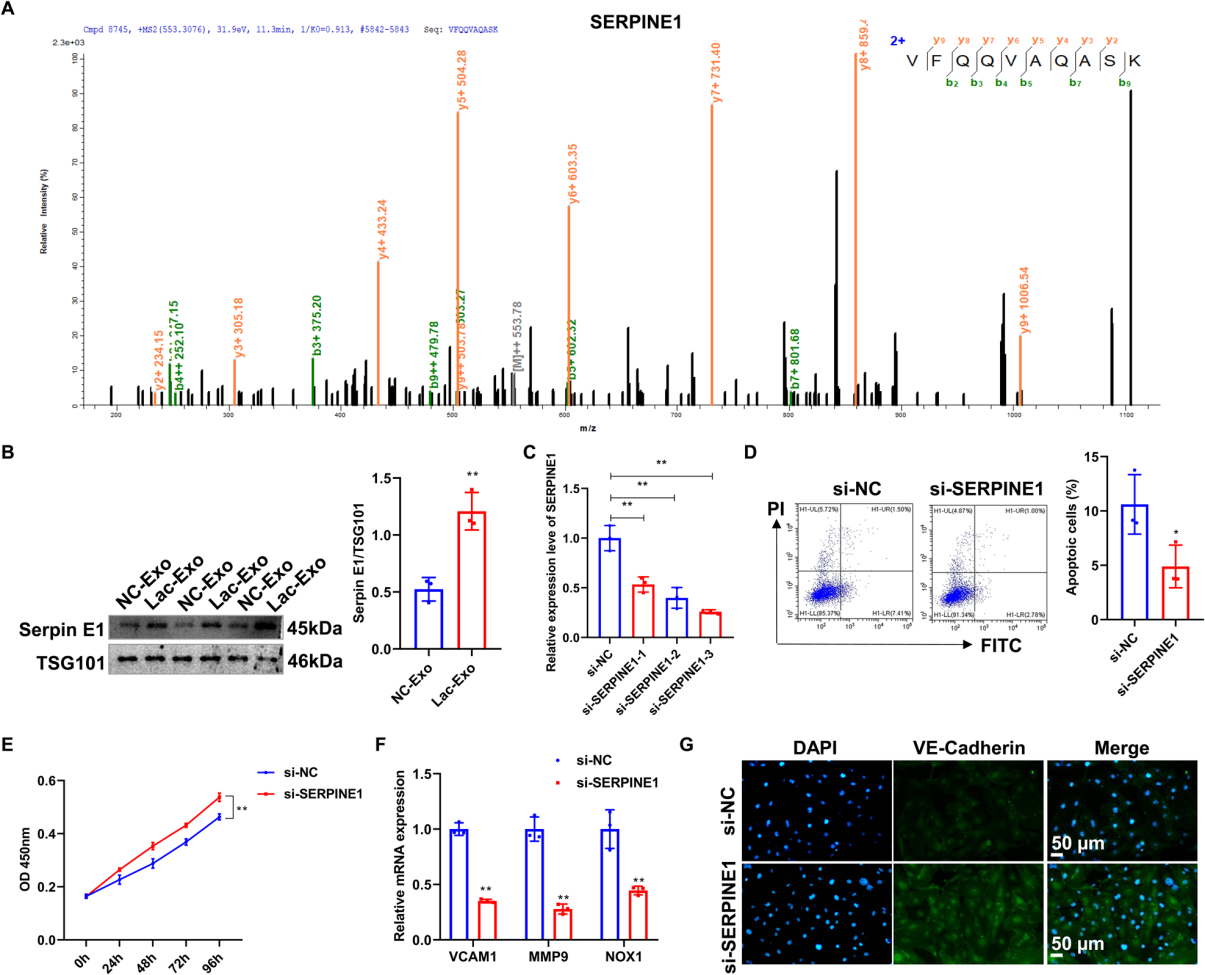
M1 macrophage-derived exosomal protein SERPINE1 induces endothelial cell dysfunction. **A** Mass spectrometry identification of SERPINE1 in exosomal proteins. **B** The expression of SERPINE1 protein in control and lactate-treated macrophage exosomes was detected by WB. **C** Knockdown efficiency of SERPINE1 in HUVECs was verified by RT-qPCR. **D** Flow cytometric analysis of apoptosis in HUVECs following SERPINE1 interference. **E** Cell viability was examined by CCK-8 assay after SERPINE1 knockdown. **F** mRNA levels of VCAM1, MMP9, and NOX1 were measured in control and SERPINE1-knockdown HUVECs using RT-qPCR. **G** VE-cadherin expression in HUVECs was evaluated by IF following SERPINE1 knockdown. Data are represented as mean ± SEM. Two-tailed Student’s t test (**B**, **D**–**F**) or one-way ANOVA (**C**). *P < 0.05, ** *P* < 0.01.

To further elucidate the molecular mechanism by which M1 macrophage-derived exosomes induce endothelial dysfunction, we isolated exosomes from both control and lactate-polarized M1 macrophages and performed mass spectrometry analysis. Combining the results of mass spectrometry with a review of the literature, we screened SERPINE1 protein, which was upregulated in the lactate group and inhibited angiogenesis, for further investigation (Fig. 9A). WB analysis confirmed its significant upregulation in M1 macrophage-derived exosomes (Fig. 9B). To assess the functional role of SERPINE1 in endothelial cells, we performed SERPINE1 knockdown in HUVECs (Fig. 9C). Flow cytometry and CCK-8 results revealed that SERPINE1 silencing markedly reduced apoptosis (Fig. 9D) and enhanced proliferation (Fig. 9E), respectively. RT-qPCR demonstrated that SERPINE1 knockdown significantly downregulated endothelial dysfunction markers, including VCAM1, MMP9, and NOX1 (Fig. 9F). Furthermore, IF staining indicated elevated VE-cadherin expression following SERPINE1 knockdown, suggesting improved endothelial barrier integrity (Fig. 9G). These findings demonstrate that M1-polarized macrophages promote endothelial dysfunction via exosomal SERPINE1.

## Discussion

**Fig. 10:**
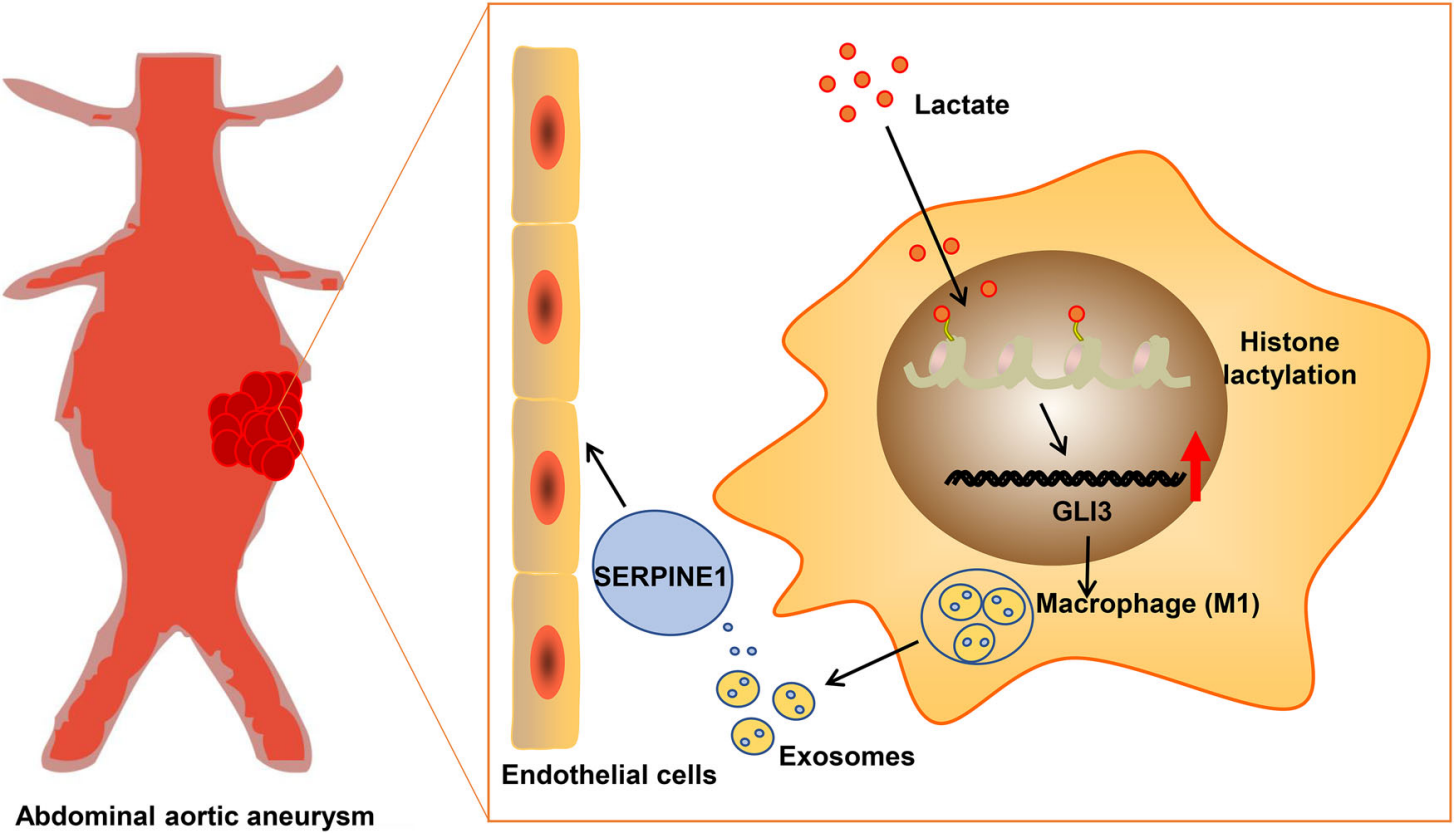
Mechanism diagram illustrating the involvement of lactylation modification in regulating macrophage M1 polarization during AAA progression. Histone lactylation-mediated GLI3 activation promotes macrophage M1 polarization, and the subsequent secretion of exosomal SERPINE1 by M1 macrophages induces endothelial dysfunction and ultimately facilitates the progression of AAA.

Macrophage polarization is known to have a pivotal role in the regulation of the formation of AAA [6]. Lactate-mediated histone lactylation promotes macrophage M1 polarization [9]. However, the mechanisms by which lactylation modification regulates M1 polarization in AAA are still unclear. In this study, we found that lactate upregulates histone lactylation levels in macrophages, which activates GLI3 signaling to promote M1 polarization. M1 macrophages participate in the progression of AAA by inducing endothelial dysfunction through the secretion of exosomal protein SERPINE1 (Fig. 10).

Single-cell RNA sequencing of the infrarenal abdominal aorta of C57BL/6 J mice showed that the resolution of inflammation and vascular repair persists throughout the development of AAA [23]. This suggests that macrophage polarization is a continuous process throughout the development of AAA. During macrophage M1 polarization, the modulation of gene expression is significantly influenced by histone lactylation [24]. Lactate promotes macrophage transition from inflammation to a reparative state by lactylating histone arginines, activating balanced gene expression, and maintaining immune homeostasis [25]. As M1 polarization leads to increased lactate production, lactylation-mediated regulation of M1 macrophage activation induces a late-stage M2-like phenotype, promoting wound healing [26]. CTF promotes M2 polarization of tumor-associated macrophages and pancreatic cancer progression by activating the IGF2BP2-CSF1/CSF1R axis through FLG-AS1/HNRNPU-mediated histone lactylation [27]. MeCP2 K271 lactylation facilitates pro-reparative M2 macrophage polarization and reduces the risk of atherosclerotic cardiovascular disease [28]. However, the potential role of lactylation-mediated M1 macrophage polarization in AAA remains unclear. This study provides the first evidence of a positive correlation between lactate-mediated histone lactylation and macrophage M1 polarization in AAA.

The GLI protein family functions as transcriptional effectors of the Hedgehog pathway, which has been shown to regulate macrophage polarization [16–18]. A study demonstrated that the collective depletion of GLI1/GLI2/GLI3 leads to increased macrophage infiltration, suggesting that nuanced alterations in Gli level have different regulatory effects on immune infiltration [29]. However, there have been no reports on the regulation of macrophage M1 polarization by GLI3. This study presents novel findings, being the first to establish that histone lactylation-mediated GLI3 promotes macrophage M1 polarization and contributes to the progression of AAA.

Increasing evidence suggests that endothelial dysfunction serves as an initial pathological event in the formation of AAA, leading to inflammation and oxidative stress in the degenerated arterial wall [20, 30]. A clinical study has shown a strong correlation between AAA and endothelial dysfunction in the elderly population [31]. Animal studies have demonstrated that endothelial dysfunction can promote the development of AAA [30, 32, 33]. Zhao et al. demonstrated that KLF11 prevents AAA by inhibiting endothelial cell dysfunction. The specific mechanism involves KLF11 suppressing the inflammatory response in cardiovascular cells, reducing the expression of MMP9, and diminishing the production of reactive oxygen species mediated by NADPH oxidase 2 in cardiovascular cells [34]. Importantly, macrophage M1 polarization plays a role in promoting endothelial cell injury [35]. There is an interaction between high glucose-induced endothelial cell inflammation and macrophage polarization [36]. Cui et al. [37] found that inflammation and endothelial dysfunction induced by M1 macrophages exacerbate coronary microvascular dysfunction. In the microenvironment of myocardial infarction, extracellular vesicles derived from pro-inflammatory M1-like macrophages inhibit angiogenesis and worsen cardiac dysfunction [13]. These findings collectively suggest that macrophage M1 polarization promotes endothelial dysfunction, which strongly supports our current demonstration that M1 macrophages induce endothelial dysfunction and accelerate AAA progression through exosome-mediated secretion of SERPINE1.

Our study does have some limitations. We elucidated the role and mechanisms by which macrophage M1 polarization regulates AAA at the cellular and animal levels. Although these results showed consistent and statistically significant trends, future studies would benefit from larger sample sizes to enhance the robustness. Furthermore, for the reliability of the conclusions, further validation is needed in AAA clinical tissue samples. Finally, the correlation between GLI3 and SERPINE1 with clinical indicators of AAA needs further exploration to provide a foundation for subsequent clinical translation.

## Conclusion

In conclusion, this study demonstrated that lactate-induced histone lactylation activates GLI3 signaling and promotes macrophage M1 polarization in AAA. The exosomal protein SERPINE1 secreted by M1 macrophages induces endothelial dysfunction, thereby driving AAA progression. These findings provide novel insights into AAA pathogenesis and identify promising molecular targets for therapeutic intervention.

## Materials and methods

### Clinical samples

We recruited nine AAA patients and 11 healthy individuals from Renji Hospital Affiliated to Shanghai Jiao Tong University School of Medicine. The inclusion criteria for AAA patients were as follows: (1) Confirmed diagnosis of AAA through imaging examinations, typically selecting patients with a diameter equal to or exceeding 3 centimeters; (2) Aged 18 or above; (3) Absence of other major illnesses such as end-stage renal disease, liver disease, cancer, etc.; (4) Patients with no history of long-term smoking or alcohol abuse. Peripheral blood samples from the participants were collected using venipuncture of the brachial vein with an anticoagulant containing EDTA.

The clinical sample used in this study was obtained in compliance with relevant regulations and ethical principles. Before they participated in this study, all participants were informed and provided their consent. This study has obtained approval from the ethics committee of Renji Hospital Affiliated to Shanghai Jiao Tong University School of Medicine.

### Lactate content detection

The Lactic Acid (L-LA) Content Assay Kit (Sangon Biotech, D799099-0100) was used to detect the lactate content in the serum of AAA patients. A volume of 100 μL of serum was taken and then combined with 1 mL of extraction solution 1. It was centrifuged at 4 °C and 12,000 × *g* for 10 minutes. Then, extraction solution 2 was added to the supernatant. The supernatant was collected after centrifugation at 12,000 × *g* for 10 minutes. The lactate content was quantified at 570 nm utilizing a spectrophotometer (Thermo Fisher, PC).

### Establishment of the AAA model

The ApoE^−/−^ C57BL/6 mice (male, 12–16 weeks) were purchased from SPF Biotech (BEIJING) to establish the model of AAA by injecting Ang II. The animal experimental protocol was reviewed and approved by Renji Hospital, Affiliated to Shanghai Jiao Tong University School of Medicine. The animal experiments described in this study adhered to the principles of the “3 R” (Reduction, Replacement, Refinement) guidelines.

The mice were randomly assigned to two groups: the control group, consisting of five mice, and the AAA group, comprising ten mice. Before the experiment, the mice were anesthetized using pentobarbital (Merck, 4390-16-3). A small incision was made on the dorsal neck of the mice. A micro-osmotic pump was placed in the subcutaneous space of the neck through the incision. Ang II (MKBio, MP5422) (1.44 mg/kg/day) was infused into the mice at a rate of 1 μg/kg per minute using the micro-osmotic pump [38]. The treatment lasted for four weeks, during which two mice in the AAA group died. The control group received a saline injection using the micro-osmotic pump. After the four-week treatment, all mice were euthanized by using an excessive amount of CO_2_, followed by cervical dislocation to confirm death. The abdominal aorta was then collected for subsequent experiments.

Throughout the study, personnel performing animal grouping were blinded to treatment allocation. All outcome assessments were conducted by researchers unaware of group assignments.

### Histological staining

The fresh abdominal aortic tissue was immersed in 4% paraformaldehyde for fixation. After dehydration and embedding, the prepared wax blocks were placed on a microtome for sectioning, with a section thickness of 4 μm. For H&E staining, dewaxed sections were stained with Harris hematoxylin and eosin to visualize nuclear and cytoplasmic structures, respectively. To evaluate extracellular matrix components, parallel sections were processed with Elastica Van Gieson (EVG) staining involving Weigert’s iron hematoxylin for nuclei, resorcin-fuchsin for elastic fibers (black), and Van Gieson’s solution for collagen (red). Complementary Masson’s trichrome staining was performed using Weigert’s hematoxylin, Biebrich scarlet-acid fuchsin for muscle fibers (red), phosphomolybdic-phosphotungstic acid differentiation, and aniline blue for collagen (blue). All stained sections were dehydrated, mounted on slides, and observed under a microscope.

### Cell culture and treatment

For the culture of THP-1 cells (Beyotime, C6960), a medium comprising RPMI-1640 (Corning, 10-040-CV) supplemented with 10% FBS and P/S (Sangon, E607011) was employed. The cells were incubated at a temperature of 37 °C, 95% air, and 5% CO_2_. To induce THP-1 cells into macrophages, logarithmic phase THP-1 cells were pelleted by centrifugation and then resuspended in RPMI-1640 medium. PMA was added to the cell suspension, and THP-1 density was adjusted to 1 × 10^5^ cells/mL. A total of 2 mL of the cell suspension was dispensed into each well of a six-well plate, and the cells were then incubated for a duration of 48 hours. Subsequently, the cells were incubated with IL-4 (20 ng/mL) and IL-13 (20 ng/mL) to induce M1 polarization.

Human umbilical vein endothelial cells HUVEC (iCell, iCell-h110) were cultured in ECM medium (iCell, PriMed-iCell-002). The cells were maintained at 37°C with 95% air and 5% CO_2_.

### Immunofluorescence (IF)

For tissue IF, the abdominal aortic tissue sections were fixed in 4% paraformaldehyde at 4 °C. Subsequently, sections were washed with 0.5% Triton X-100/3% BSA. Primary antibodies against CD86 (Invitrogen, PA5-88284) and H3K18la (Jingjie PTM Biolab, PTM-1406RM) were incubated with the sections at 37°C for one hour. After washing with 3% BSA, the sections were incubated with the secondary antibody Goat Anti-Mouse IgG H&L (HRP) (Abcam, ab205719). The fluorescence signals were observed under a fluorescence microscope.

For cell IF, after fixation and blocking, primary antibodies against CD86 (Invitrogen, PA5-88284), CD206 (Invitrogen, PA5-101657), and VE-Cadherin (CST, 2500S) were added to each well and incubated overnight at 4°C. Then, the cells were subjected to incubation with the Cy3-conjugated goat anti-rabbit IgG (H + L) secondary antibody (Beyotime, A0516) or goat anti-rabbit Alexa Fluor 488 secondary antibody (Abcam, ab150077). DAPI staining was performed for 10 minutes in the dark, followed by PBS washing. A fluorescence microscope was used to visualize the fluorescence signals.

### IF and fluorescence in situ hybridization (IF/FISH)

To assess the colocalization of histone lactylation (H3K18la) and GLI3 DNA in macrophages, we performed a combined IF and FISH assay. Macrophages were fixed with 4% paraformaldehyde (Beyotime, P0099) for 20 minutes, washed, and optionally treated with RNase. Cells were permeabilized with 0.5% Triton X-100, followed by graded ethanol dehydration. GLI3 DNA probes were denatured at 75 °C for 5–8 minutes, rapidly cooled, and hybridized overnight at 37 °C. Post-hybridization washes were performed using SSC buffers at elevated temperatures. For immunostaining, samples were blocked with 3% BSA or 5% serum, incubated with primary antibodies against H3K18la (Jingjie PTM Biolab, PTM-1406RM) at 4 °C overnight, followed by fluorophore-conjugated secondary antibodies Goat Anti-Rabbit IgG H&L (Alexa Fluor® 488)(Abcam, ab150077) at room temperature for 1 hour. Nuclei were counterstained with DAPI. Finally, samples were mounted with an antifade solution and imaged using fluorescence.

### siRNA transfection

To knockdown GLI3 in macrophages and SERPINE in HUVECs, Lipofectamine™ 2000 transfection reagent (Invitrogen) was utilized for the transfection of siRNA (GenePharma, Shanghai, China). The diluted siRNA and transfection reagent were mixed and incubated for 15 minutes. After incubation, the 100 μL mixture was added to the macrophages or HUVECs. Two days after transfection, the knockdown efficiency was evaluated. The siRNA sequences employed are provided in the Supplementary Table 1.

### Western blot (WB)

Protein lysis buffer (Thermo) was used to lyse macrophages and precipitate exosomes, followed by subjecting them to SDS-PAGE gel electrophoresis. A PVDF membrane was used for the transfer of the SDS-PAGE gel. The membrane was incubated with specific antibodies against Histone H3 (Arigo, ARG54767, 1:2000 dilution), H3K18la (PTMBIO, PTM-1406RM, 1:2000 dilution), GAPDH (Proteintech, 60004-1-Lg, 1:2000 dilution), GLI3 (Proteintech, 28272-1-AP, 1:1000 dilution), Serpin E1 (Proteintech, 13801-1-AP, 1:1000 dilution), Alix (Proteintech, 12422-1-AP, 1:15000 dilution), CD9 (Proteintech, 20597-1-AP, 1:2000 dilution), CD63 (Proteintech, 25682-1-AP, 1:1000 dilution), and Tsg101 (Proteintech, 67381-1-lg, 1:10000 dilution) for 3 hours. Subsequently, the membrane was incubated with secondary antibodies, either Goat Anti-Mouse IgG H&L (HRP) (Abcam, ab205719, 1:1000 dilution) or Goat Anti-Rabbit IgG H&L (HRP) (Abcam, ab6721, 1:20000 dilution), for 2 hours. The membrane was then visualized using a high-sensitivity ECL chemiluminescence kit (Thermo).

### RNA extraction and reverse transcription quantitative polymerase chain reaction (RT-qPCR)

Trizol (Invitrogen) was utilized for the extraction of total RNA from macrophage cells. A reverse transcription kit (Thermo, K1622) was employed for cDNA synthesis. The reaction program included incubation at 25 °C for 5 minutes, 42 °C for 6 minutes, and 70 °C for 5 minutes. Real-time PCR amplification of the obtained cDNA was carried out using the 2× Master Mix kit (Roche) on a real-time fluorescence quantitative PCR machine (Applied Biosystems Inc., ABI Q6). The reaction program commenced with an initial denaturation step at 95 °C for 10 minutes, followed by 45 cycles of denaturation at 95 °C for 15 seconds and annealing at 60 °C for 60 seconds. GAPDH was used as an endogenous control. The primer sequences for all genes can be found in Supplementary Table 1. The 2^-ΔΔCT^ method was employed to calculate the relative expression of the target gene.

### ELISA

The IL-1β, TNF-α, and IL-10 levels were quantified using the IL-10 ELISA Kit (Enzyme-linked Biotechnology, ml027436), IL-1β ELISA Kit (Enzyme-linked Biotechnology, ml027417), and TNF-α ELISA Kit (Enzyme-linked Biotechnology, ml077385), respectively. The macrophages underwent centrifugation at 1000 × *g* for 20 minutes. Each well of the reaction plate received a 50 μL volume of diluted standard or test sample. Following the addition of 50 μL of biotinylated antibody, the mixture was incubated at 37 °C for 1 hour. After adding 80 μL of streptavidin-HRP, the mixture was incubated at 37 °C for 30 minutes. In each well, 50 μL of substrate A and 50 μL of substrate B were added, followed by incubation at 37 °C in the dark. The OD values of each well were immediately recorded at a wavelength of 450 nm.

### Transcriptome sequencing

Healthy human blood was collected, and PBMC cells were isolated using lymphocyte fractionation solution (Ficoll). The PBMCs were subjected to a six-day treatment with 50 ng/mL of M-CSF to stimulate their differentiation into macrophages. During the incubation, lactate (10 mM) was introduced to the macrophages for 24 hours. Trizol (Invitrogen) was employed for the extraction of total RNA from macrophages, and mRNA libraries were constructed for sequencing utilizing the Illumina HiSeq. The raw sequencing data obtained underwent quality control with the aid of FastQC software to obtain clean reads. Then, alignment of the clean reads was performed against the reference genome sequence. The expression values of genes were standardized using RPKM. The DESeq algorithm was employed for the analysis of differential gene expression. In the screening process, genes with a Log2FC > 1 or < −1, and a false discovery rate (FDR) < 0.05, were identified as differentially expressed genes (DEGs).

The Gene Ontology (GO) database was utilized for performing GO annotation of DEGs. To determine the significance of each GO term, Fisher’s test was employed to calculate the corresponding *p* value. Similarly, the DEGs were subjected to analysis utilizing the Kyoto Encyclopedia of Genes and Genomes (KEGG) database to identify significant pathways. The significance criteria for screening were set as *p* value < 0.05.

### CUT&Tag

Healthy human blood was collected to isolate PBMCs. The PBMCs were then treated with 50 ng/mL of M-CSF for six days, resulting in the differentiation of macrophages. The macrophages were subjected to a 24-hour incubation period with lactate (10 mM). The cells were subjected to incubation with magnetic beads for 10 minutes, and then H3K18la antibody (PTMBIO, PTM-1406RM) was added to enrich genes with histone lactylation modification. CUT&Tag libraries were generated, and the Illumina HiSeq platform was employed for sequencing. The SEACR package was used for enrichment region identification (Peak Calling). The ChIPseeker software was used to annotate the peaks identified by CUT&Tag, thus identifying regions where histone lactylation occurs. The MEME software was utilized to perform motif analysis on the obtained peaks. The MAnorm2 software was used for differential peak identification. GO/KEGG analysis was conducted to functionally annotate the differentially modified genes obtained through the screening process.

### Chromatin immunoprecipitation combined with qPCR (ChIP-qPCR)

ChIP-qPCR was performed using the EZ-Magna ChIP™ A/G Chromatin Immunoprecipitation Kit (Millipore, 17-10086) to analyze protein-DNA interactions in macrophages. Cells were crosslinked with 1% formaldehyde (Merck, F8775-500ML) for 10 minutes at room temperature, quenched with glycine, and lysed using cell lysis and nuclear lysis buffers containing protease inhibitors. Chromatin was sheared to 100–400 bp fragments using a Bioruptor sonicator (8 cycles of 30 sec on/off pulses). After pre-clearing, 300 μL of chromatin was immunoprecipitated overnight at 4 °C with 5 μg anti-H3K18la antibody (Jingjie PTM Biolab, PTM-1427RM) or 1 μg rabbit IgG control antibody coupled to protein A/G magnetic beads. Beads were washed sequentially with low salt, high salt, LiCl, and TE buffers. Immunoprecipitated DNA was eluted, reverse crosslinked, and purified by phenol-chloroform extraction followed by ethanol precipitation. Purified DNA was resuspended in Tris-HCl (pH 8.0) and analyzed by qPCR, with input chromatin samples serving as normalization controls.

### Dual-luciferase reporter gene assay

Full-length wild-type (WT) and mutant (MUT, with deletion of the H3K18la modification site) GLI3 sequences were synthesized and cloned into the pmirGLO dual-reporter vector (Promega) using XhoI/NotI restriction sites. For transfection, 293 T cells were seeded in 96-well plates and transfected with 100 ng of either pmirGLO-GLI3-WT or pmirGLO-GLI3-MUT plasmid using Lipofectamine 2000 reagent (Invitrogen). Cells were divided into four treatment groups: (1) lactate-treated GLI3-WT, (2) untreated GLI3-MUT, (3) untreated GLI3-WT, and (4) lactate-treated GLI3-MUT. After 48 hours of incubation, cells were lysed and luciferase activity was measured using the Dual-Luciferase Reporter Gene Assay Kit (Yeasen). Firefly luciferase activity values were normalized to Renilla luciferase activity for data analysis.

### Exosome isolation

For exosome isolation, macrophages were divided into control and lactate-treated (10 mM sodium lactate, 24 hours) groups, followed by 48 hours culture in serum-free medium to allow exosome secretion. The supernatant was subjected to sequential centrifugation: 300 × *g* for 10 minutes to remove intact cells, 2000 × *g* for 10 minutes to eliminate dead cells, and 10,000 × *g* for 30 minutes (all at 4 °C) to clear cellular debris. The clarified supernatant was then filtered through 0.22-μm syringe filters before final ultracentrifugation at 120,000 × *g* for 2 hours at 4 °C using a HITACHI himac CPBOWX ultracentrifuge. The resulting exosome pellet was carefully resuspended in 200 μL ice-cold 1× PBS with extensive gentle pipetting (>200 mixing cycles) to ensure complete dissolution, and aliquots were stored at 4 °C for immediate use or −80 °C for long-term preservation. This rigorous protocol, performed under strictly controlled temperature conditions and incorporating multiple purification steps, ensures the isolation of high-purity exosomes suitable for downstream characterization and functional studies.

#### Transmission electron microscopy (TEM)

TEM was performed to characterize exosomes isolated from macrophages. Briefly, 5–10 μL of purified exosome samples were applied to copper grids and allowed to settle for 1 minute at room temperature. Excess liquid was carefully removed using filter paper. Samples were then washed with PBS to remove contaminants, followed by negative staining with 10 μL phosphotungstic acid for 1 minute. After removing the staining solution, grids were air-dried for 2 minutes at room temperature. Imaging was conducted using a transmission electron microscope (JEOL, JEM-1200EX) operating at 100 kV accelerating voltage. Representative micrographs were captured to assess exosome morphology and size distribution.

### Tube formation assay

To allow solidification, 200 μL of Matrigel (356230, Corning) was added to each well of a 24-well plate, followed by incubation at 37 °C for 1 hour. Then, 8 × 10^4^ HUVEC cells were seeded onto the solidified Matrigel. The conditioned medium of macrophages and lactate-treated macrophages, mixed with the ECM complete medium, was incubated with the cells in a CO_2_ incubator for 24 hours. The 24-well plate was then removed, and photographs of the formed vessels were captured using an inverted phase-contrast microscope.

### CCK-8 assay

A CCK-8 assay kit (Beyotime) was employed to assess the viability of HUVEC cells. A total of 100 µL of HUVEC cells were seeded per well in a 96-well plate, resulting in a density of 3000 cells per well. The plate was placed in a 37 °C incubator with 5% CO_2_ overnight. Then, each well received an addition of 10 µL of CCK-8 solution. A microplate reader (Infinite M1000, TECAN) was utilized to measure the absorbance at 450 nm after a 2-hour incubation.

### Flow cytometry

To investigate the effect of SERPINE1 on HUVEC apoptosis, we performed an Annexin V-FITC/PI double staining assay using a commercial apoptosis detection kit (E606336-0100, Sangon Biotech). Briefly, HUVECs with SERPINE1 knockdown were collected and stained with 5 μL Annexin V-FITC for 15 minutes at room temperature in the dark. After washing with PBS, cells were centrifuged at 1000 rpm for 5 minutes, and the resulting pellet was resuspended in 190 μL of 1× Binding Buffer. Immediately before flow cytometry analysis, 10 μL Propidium Iodide (PI) was added to each sample, and all measurements were completed within four hours of staining to ensure optimal fluorescence signals.

### Statistical analysis

All experiments were conducted using a minimum of three biological replicates. GraphPad Prism 8 was utilized for statistical analysis. The mean values, along with their corresponding standard deviations (SD), were used to present the results. Prior to parametric testing, normality was confirmed using the Shapiro–Wilk test. For data conforming to normal distribution, a *t* test was employed to compare two groups, and one-way ANOVA followed by Tukey’s test was used to compare multiple groups. Statistical significance was defined as a *p* value <0.05.

## Supplementary information

Supplementary Table 1

WB raw gel

## Data Availability

The data presented in the study (RNA-seq and CUT&Tag raw data) have been deposited in the NCBI repository under accession numbers (PRJNA1299899) and (PRJNA1300311).
