# Supplementary material for: Histone lactylation-induced GLI3 activation drives macrophage M1 polarization and exosomal SERPINE1 release in abdominal aortic aneurysm progression
Source: Cell Death Discov. 2025 Nov 10;11:523. doi: 10.1038/s41420-025-02748-7 (PMC12603228; doi:10.1038/s41420-025-02748-7)
Supplement: Supplementary file 1 — Supplementary Table 1 [file 41420_2025_2748_MOESM1_ESM.docx]

**Supplementary Table 1**: Primer and siRNA fragments sequences

| Gene | Sequence |
| --- | --- |
| CD80 | Forward: 5’- ACCTGGCTGAAGTGACGTTA -3’  Reverse: 5’- GAGAGGTGAGGCTCTGGAAA -3’ |
| iNOS | Forward: 5’- AGGGACAAGCCTACCCCTC -3’  Reverse: 5’- CTCATCTCCCGTCAGTTGGT -3’ |
| MRC2 | Forward: 5’- CTCTGGATTGGGCTGGCT -3’  Reverse: 5’- GGCCCCGTCCACATCTAC -3’ |
| Arg1 | Forward: 5’- TGGACAGACTAGGAATTGGCA -3’  Reverse: 5’- CCAGTCCGTCAACATCAAAACT -3’ |
| MCP1 | Forward: 5’- CAGCCAGATGCAATCAATGCC -3’  Reverse: 5’- TGGAATCCTGAACCCACTTCT -3’ |
| CD163 | Forward: 5’- GAAGACAGAGACAGCGGCTT -3’  Reverse: 5’- GGTATCTTAAAGGCTCACTGGGT -3’ |
| COL27A1 | Forward: 5’- CAAGAGGGGCAAGATGGGTA-3’  Reverse: 5’- TCGGACCCAACACTCCTAAG-3’ |
| SLFNL1 | Forward: 5’- AGGTGTCAGAGCCCTTCATG-3’  Reverse: 5’- CCGCCTCACCACTTCAATG-3’ |
| GLI3 | Forward: 5’- TCATGAGGGCCGTTACCATT-3’  Reverse: 5’- AATGTAGGGATGTGGAGGGC-3’ |
| SEMA5A | Forward: 5’- GCCGTGTGTGTTTGACTCTA-3’  Reverse: 5’- ATAGACGAGCAGGGTGAGGA-3’ |
| SERPINE1 | Forward: 5’- AGTGGACTTTTCAGAGGTGGA-3’  Reverse: 5’- GCCGTTGAAGTAGAGGGCATT-3’ |
| GAPDH | Forward: 5’- ACAACTTTGGTATCGTGGAAGG -3’  Reverse: 5’- GCCATCACGCCACAGTTTC -3’ |
| GLI3-siRNA-1 | Forward: 5’- ACGCAAUCACUAUGCAGCCACAGAA-3’  Reverse: 5’- UUCUGUGGCUGCAUAGUGAUUGCGU -3’ |
| GLI3-siRNA-2 | Forward: 5’- GGACCAAAUGGAUGGAGCACGUAAA-3’  Reverse: 5’- UUUACGUGCUCCAUCCAUUUGGUCC -3’ |
| GLI3-siRNA-3 | Forward: 5’- CCGCAGUAUGGGAACUGUCUCAACA-3’  Reverse: 5’- UGUUGAGACAGUUCCCAUACUGCGG -3’ |
| SERPINE1-siRNA-1 | Forward: 5’- GCAGCAGAUUCAAGCAGCUAUGGGA-3’  Reverse: 5’- UCCCAUAGCUGCUUGAAUCUGCUGC -3’ |
| SERPINE1-siRNA-2 | Forward: 5’- GAGAACCUGGGAAUGACCGACAUGU -3’  Reverse: 5’- ACAUGUCGGUCAUUCCCAGGUUCUC -3’ |
| SERPINE1-siRNA-3 | Forward: 5’- CAGACAGUUUCAGGCUGACUUCACG -3’  Reverse: 5’- CGUGAAGUCAGCCUGAAACUGUCUG -3’ |
| si-NC | Forward: 5’- UUCUCCGAACGUGUCACGUTT-3’  Reverse: 5’- ACGUGACACGUUCGGAGAATT -3’ |
