# Supplementary material for: Histone lactylation-induced GLI3 activation drives macrophage M1 polarization and exosomal SERPINE1 release in abdominal aortic aneurysm progression
Source: Cell Death Discov. 2025 Nov 10;11:523. doi: 10.1038/s41420-025-02748-7 (PMC12603228; doi:10.1038/s41420-025-02748-7)
Supplement: Supplementary file 2 — WB raw gel [file 41420_2025_2748_MOESM2_ESM.pptx]

## Slide 1
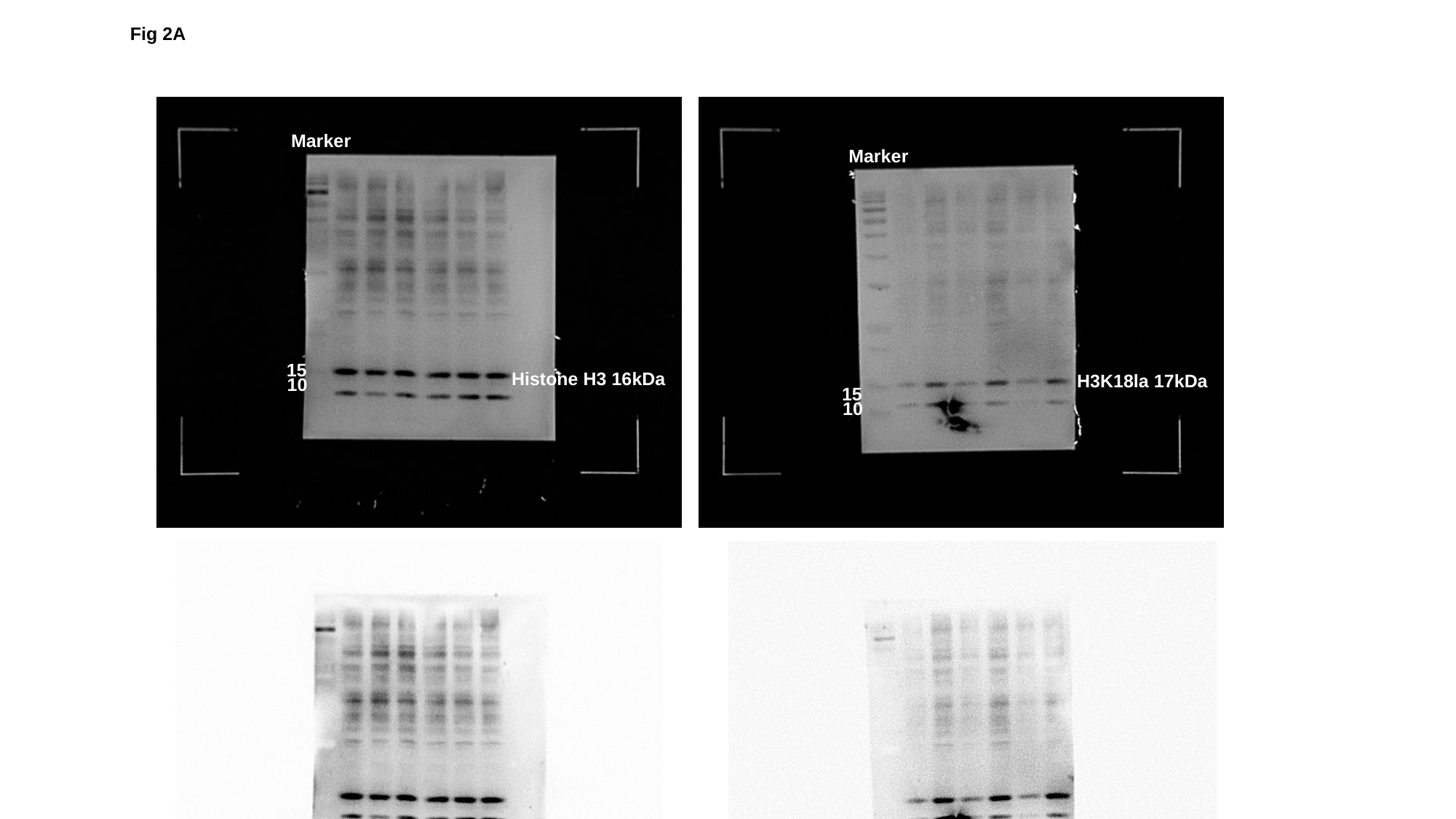

Fig 2A
Marker
Marker
15
Histone H3 16kDa
H3K18la 17kDa
10
15
10

## Slide 2
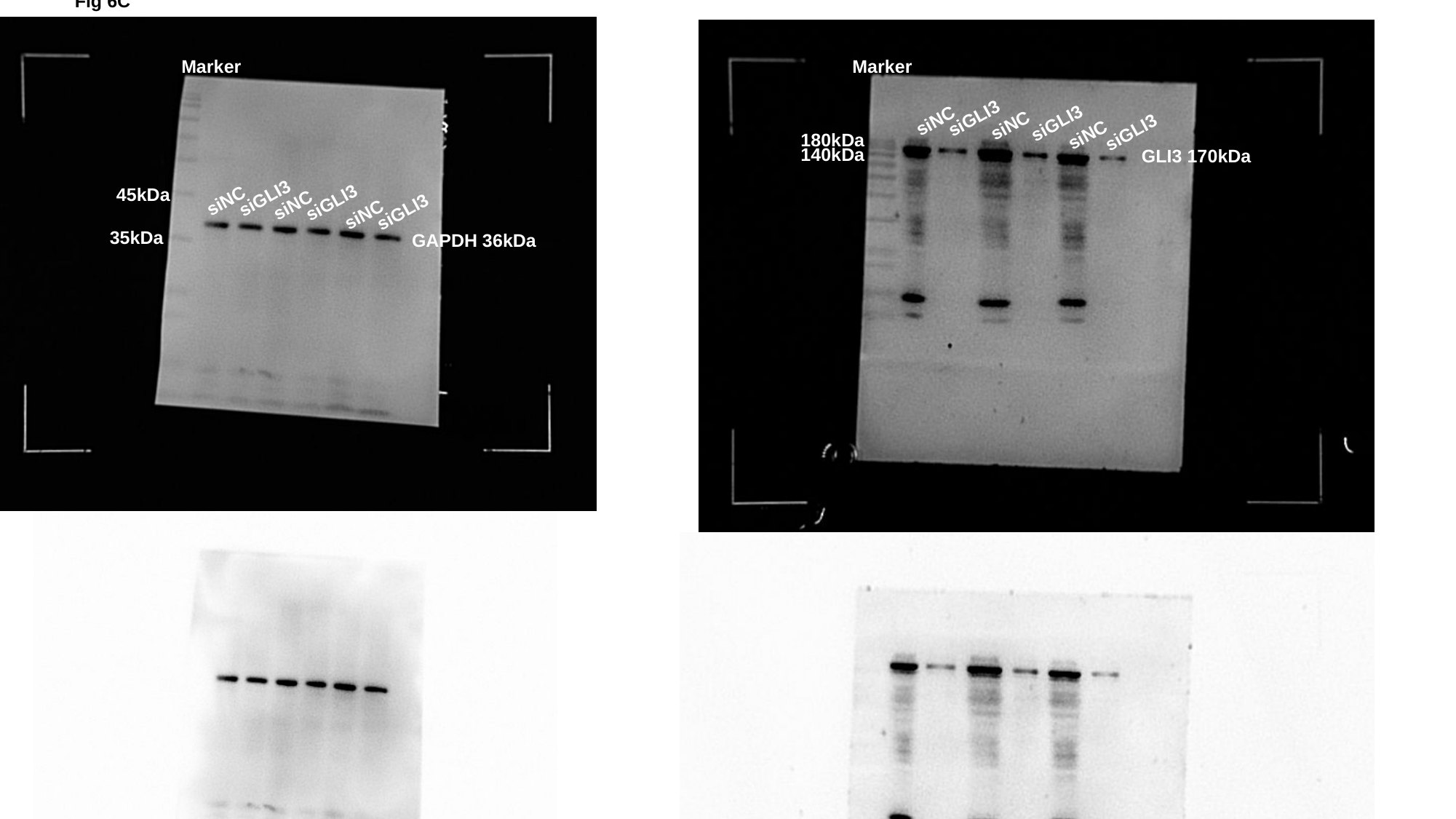

Fig 6C
Fig 6C
Marker
Marker
siGLI3
siNC
siGLI3
siNC
siGLI3
siNC
180kDa
140kDa
GLI3 170kDa
45kDa
siGLI3
siNC
siGLI3
siNC
siGLI3
siNC
35kDa
GAPDH 36kDa

## Slide 3
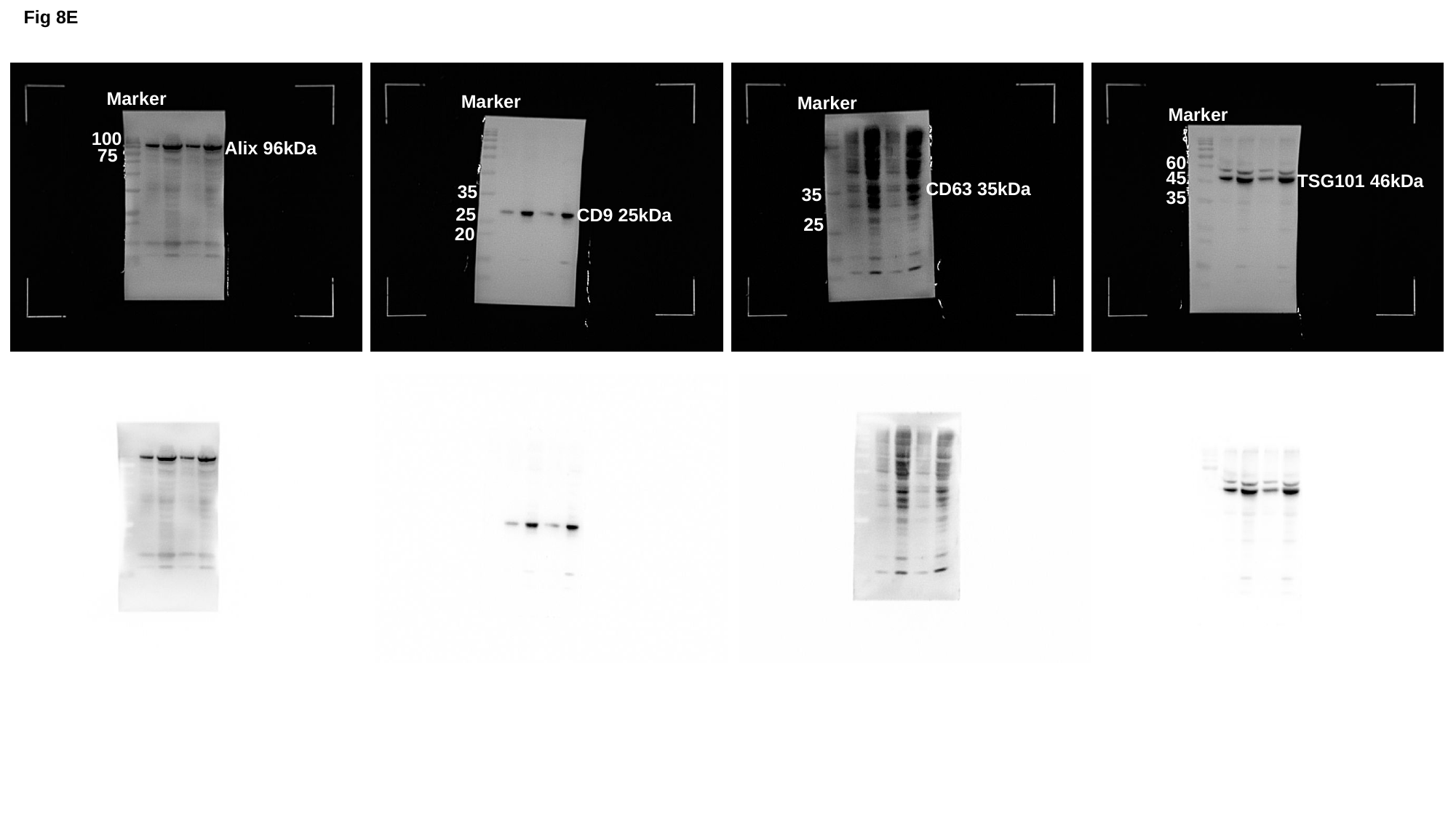

Fig 8E
Marker
Marker
Marker
Marker
100
Alix 96kDa
75
60
45
TSG101 46kDa
CD63 35kDa
35
35
35
25
CD9 25kDa
25
20

## Slide 4
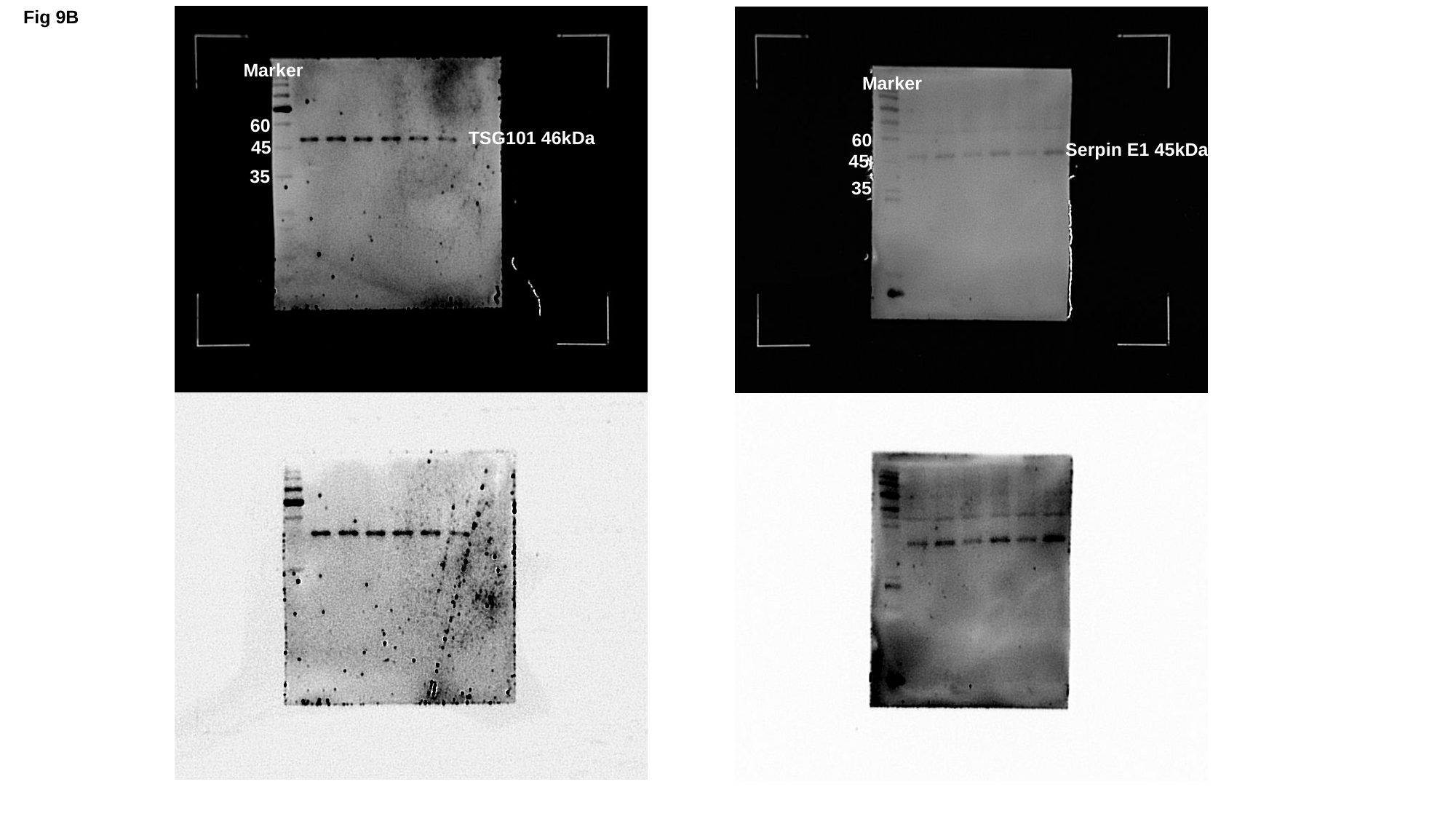

Fig 9B
Marker
Marker
60
TSG101 46kDa
60
45
Serpin E1 45kDa
45
35
35
